# Wnt/β-Catenin Signaling: The Culprit in Pancreatic Carcinogenesis and Therapeutic Resistance

**DOI:** 10.3390/ijms20174242

**Published:** 2019-08-30

**Authors:** Monish Ram Makena, Himavanth Gatla, Dattesh Verlekar, Sahithi Sukhavasi, Manoj K. Pandey, Kartick C. Pramanik

**Affiliations:** 1 Department of Physiology, Johns Hopkins School of Medicine, Baltimore, MD 21205, USA; 2 Department of Pediatric Oncology, Johns Hopkins School of Medicine, Baltimore, MD 21287, USA; 3 Department of Cell Biology and Biochemistry, Texas Tech University Health Sciences Center, Lubbock, TX 79430, USA; 4 Center for Distance Learning, GITAM University, Visakhapatnam 530045, India; 5 Department of Biomedical Sciences, Cooper Medical School of Rowan University, Camden, NJ 08103, USA; 6 Department of Basic Sciences, Kentucky College of Osteopathic Medicine, University of Pikeville, Pikeville, KY 41501, USA

**Keywords:** pancreatic cancer, Wnt/β-catenin signaling pathway, Wnt pathway inhibitors, therapeutic resistance

## Abstract

Pancreatic ductal adenocarcinoma (PDAC) is responsible for 7.3% of all cancer deaths. Even though there is a steady increase in patient survival for most cancers over the decades, the patient survival rate for pancreatic cancer remains low with current therapeutic strategies. The Wnt/β-catenin pathway controls the maintenance of somatic stem cells in many tissues and organs and is implicated in pancreatic carcinogenesis by regulating cell cycle progression, apoptosis, epithelial-mesenchymal transition (EMT), angiogenesis, stemness, tumor immune microenvironment, etc. Further, dysregulated Wnt has been shown to cause drug resistance in pancreatic cancer. Although different Wnt antagonists are effective in pancreatic patients, limitations remain that must be overcome to increase the survival benefits associated with this emerging therapy. In this review, we have summarized the role of Wnt signaling in pancreatic cancer and suggested future directions to enhance the survival of pancreatic cancer patients.

## 1. Introduction

### 1.1. Pancreatic Cancer

The exocrine pancreas contains acinar and duct cells and secretes digestive enzymes. This constitutes the majority (more than 95%) of the pancreatic mass. The pancreatic juice from the exocrine pancreas is supplied by the pancreatic duct to the bile duct and aids in digestion. The endocrine pancreas (the islets) makes up 1%–2% of the pancreatic mass. The islets include alpha and beta cells and secrete insulin and glucagon into the blood [1]. More than 85% of pancreatic cancer cases are diagnosed as pancreatic ductal adenocarcinoma (PDAC). PDAC develops from the exocrine cells. Other pancreatic neoplasms include neuroendocrine tumors and acinar carcinomas, colloid carcinomas, pancreatoblastomas, and solid-pseudopapillary neoplasms [2].

About 7.3% of all cancer deaths are attributed to PDAC. The 5-year survival in patients with PDAC is approximately 8.5% in the USA. Though there is a steady increase in survival for most cancers over the decades, the pancreatic cancer survival rate does not follow a similar trend with current treatment strategies [2,3]. New PDAC cases are rising at an average of 0.5% annually for the last decade. In 2018, in the United States, 55,440 new pancreatic cancer cases were diagnosed. By 2030, PDAC is projected to cause the second highest cancer-related mortality rate in the US [3,4,5]. Globally, there is a similar trend in pancreatic cancer mortality, emphasizing the universal need to develop novel strategies to treat PDAC [6].

Risk factors for pancreatic cancer include late-onset, metastatic disease presentation, family history (~15% of the PDAC cases carry mutations in *BRCA2, BRCA1, CDKN2A, ATM, STK11, PRSS1, MLH1,* and *PALB2*), genetic alterations (RAS oncogene is mutated in 90% of cases), cigarette smoking, and inactivating mutations (*TP53, CDKN2A, SMAD4 ARID1A, MLL3, and TGFBR2*) [2]. In-effective treatment strategies are one of the major factors for low survival rates in pancreatic cancer. At initial diagnosis, fewer than 20% of PDAC patients are qualified for surgery. Further, 90% of patients relapse and succumb to death after surgery. In spite of this, surgery is the only curative option [7]. The FOLFIRINOX regimen (oxaliplatin, folinic acid, irinotecan, bolus fluorouracil, infusional fluorouracil) or a combination of gemcitabine and nanoparticle albumin-bound paclitaxel are considered standard treatments for patients with PDAC. Due to the side effects of these chemotherapeutic drug cocktails, only 30%–40% of PDAC patients are eligible for treatment [2,7]. Even though immunotherapy has revolutionized cancer treatment, PDAC does not respond to immune checkpoint blockade. This may be due to a low mutation burden, with a subsequent paucity in T-cell infiltration and the development of an immune-suppressive tumor microenvironment [8]. These facts highlight the need to extend our understanding of the pathways regulating PDAC oncogenesis, to develop novel therapeutic strategies, to improve the long-term survival of PDAC patients.

High-throughput sequencing of the human PDAC exome revealed that tumors have, on average, 63 genetic mutations, including some high-frequency mutations and a much larger number of heterogeneous lower-frequency mutations [9]. Interestingly, these highly variable alterations form a cluster of 12 signaling pathways, which include the Wnt signaling pathway [9].

### 1.2. Wnt/β-Catenin Signaling Pathway

**Figure 1:**
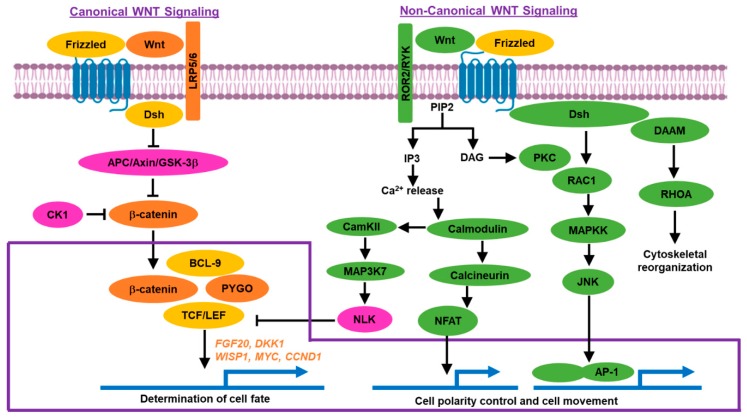
Canonical (β-catenin dependent) and non-canonical (β-catenin independent) Wnt signaling pathways.

The *Wnt1* gene was first identified by mutagenesis screening for developmental patterns in *Drosophila melanogaster* during the early 1980s. Subsequent genetic screens identified other members of the Wnt family [10]. Wnt signaling pathway regulates diverse functions, such as embryonic development, cell polarity, proliferation, migration, survival, and maintenance of somatic stem cells [11,12]. Due to its involvement in key functions, dysregulation of the Wnt pathway is implicated in many human diseases [10,13]. Components of the Wnt pathway include secreted glycoproteins, the frizzled family of transmembrane receptors, the lipoprotein receptor-related protein (LRP) family of co-receptors, and other downstream components. Canonical (β-catenin dependent) and non-canonical (β-catenin independent) pathways are the two main Wnt signaling pathways (Figure 1) [14].

#### 1.2.1. Canonical Pathway 

Signaling via the canonical pathway inhibits the degradation of β-catenin, which in turn regulates the transcription of several genes. Wnt ligand is a secreted glycoprotein, which requires lipid modification. It is acylated by a porcupine, a membrane-bound O-acyltransferase located in the endoplasmic reticulum. Wnt binds to a frizzled-related family of proteins, leading to the formation of a larger cell surface complex with LRP5/6. E3 ubiquitin-protein ligases ZNRF3 and RNF43 can act as negative regulators of the Wnt pathway by degrading Wnt receptor complex components frizzled and LRP6. The activity of ZNRF3 and RNF43 can be inhibited by R-spondin. Wnt antagonist Dickkopf-1 (DKK1) can also prevent Wnt ligand from forming a complex with LRP5/6 receptors. In the absence of the Wnt ligand, constitutively expressed β-catenin is phosphorylated by CK1 and the APC/Axin/GSK-3β-complex, leading to ubiquitylation and proteasomal degradation of β-catenin [10,14,15]. Wnt ligands, acting either through autocrine or paracrine signaling, bind to the frizzled receptors, which cooperate with LRP5/6 co-receptors, to initiate a phosphorylation cascade that activates disheveled (Dsh). This permits disassociation of the β-catenin degradation complex APC/Axin/GSK-3β, which allows translocation of β-catenin across the nuclear membrane. β-catenin then binds to the TCF/LEF family of transcription factors and activates the transcription of target genes and coactivators of transcription, such as the binding protein of the cAMP response element-binding protein (CBP, CREB binding protein), E1A-associated protein p300, Pygopus (PYGO), BCL-9, and Brahma-related gene 1 (BRG1). Apart from TCF/LEF binding, β-catenin also activates transcription through association with the FOXO family of transcription factors [10,14,15].

#### 1.2.2. Non-Canonical Pathway 

Non-canonical Wnt signaling involves two pathways, planar cell polarity (PCP) pathway and Wnt/Ca^2+^ pathway. In the PCP pathway, Wnt binds to frizzled transmembrane receptors and activates Dsh at the cell membrane. Dsh activates small GTPases RAC1 and Ras homolog gene family member A (RHOA), which in turn activates the RhoA-Rho-associated kinase axis (ROCK) and c-Jun N-terminal kinase (JNK). This pathway is known to exert effects on cell polarity and cytoskeleton organization [16,17].

Calcium is a crucial factor in many key cellular processes [18,19]. In the Wnt/Ca^2+^ pathway, frizzled receptors mediate the activation of heterotrimeric G proteins, causing calcium release from the endoplasmic reticulum. Elevated Ca^2+^ levels activate calcium-binding proteins, including protein kinase C (PKC), calcineurin, and calmodulin-dependent kinase II (CamKII). These components trigger dephosphorylation of the transcription factor NFAT, resulting in nuclear translocation and the subsequent regulation of various genes that control cell fate and cell migration [16,17].

## 2. Wnt/β-Catenin Signaling in Pancreatic Cancer

**Figure 2:**
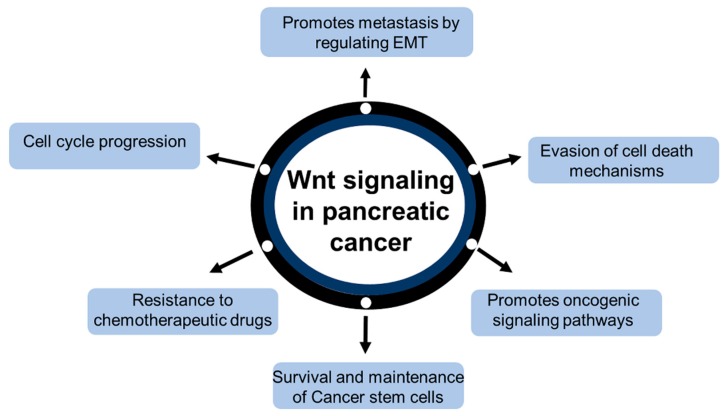
Oncogenic role of Wnt/β-catenin signaling in pancreatic cancer.

Microarray analysis of 226 PDAC samples and 65 normal pancreatic tissue samples showed that Wnt and P53 signaling pathways played an important role in PDAC oncogenesis. Protein-protein interaction network analysis revealed that DKK1 and HMGA2 were hub genes, each having a high degree of connectivity. DKK1 and HMGA2 are strongly associated with WNT3A and TP53 separately [20]. The Wnt signaling pathway is highly implicated in pancreatic carcinogenesis (Figure 2). Numerous ligands, receptors, and secondary messengers converge in the nuclear translocation of β-catenin, which transcribes genes, such as cyclin D1, cyclin E, MMP-7, c-myc, VEGF, and others. These genes are involved in various hallmarks of cancer, such as cell cycle progression, epithelial-mesenchymal transition (EMT), and angiogenesis. Also, the Wnt signaling pathway has been reported to promote resistance to apoptosis and maintenance of cancer stem cells, leading to the pathogenesis of pancreatic cancer [21]. Increased expression of canonical Wnt ligands, such as Wnt2 [22], Wnt5a [23], and Wnt7a’s [24], have been observed in pancreatic cancer tissues, along with persistent activation of the Wnt pathway, leading to cancer progression.

In addition to the above-mentioned canonical ligands, the Wnt pathway is also activated by various non-canonical ligands. Activation of Wnt pathway by non-canonical ligands, such as GATA6 [25], R-spondin [26], R-spondin2 [27], cullin 4B (CUL4B) [28], CDK8, K-ras [29], Rab5a and Rab11a [30,31], and MUC1 and MCU4 [32,33], leads to a progression of pancreatic cancer. Furthermore, K-ras signaling increases the interaction of β-catenin with CBP [34], which is a histone acetyltransferase known to increase gene expression by acetylating histones [35]. Furthermore, CBP also acetylates various transcription factors, modulating their activity [36,37]. It has also been reported that hypoxic conditions aid in pancreatic tumor progression [38]. Further, hypoxic conditions decrease the cytotoxic potential of anti-cancer agents [39]. Hypoxic conditions in pancreatic tumors stabilize HIF-2α, which forms a complex with β-catenin, increasing β-catenin activity while favoring tumor progression [38]. Besides, HIF-2α transcribes and maintains the levels of β-catenin and Smad4 in pancreatic tumors cells [40]. In comparison to normal, pancreatic cancer tissues have decreased levels of Fbxw7 [41], merlin [42], and klotho [43], which all suppress β-catenin levels, resulting in elevated β-catenin levels and activity. Proteasomal degradation of β-catenin is regulated by its phosphorylation and ubiquitination. Zinc finger protein 281 (ZNF 281), whose levels are upregulated in pancreatic cancer, decreases the ubiquitination of β-catenin and activates Wnt pathway [44]. Similarly, the leucine-rich flightless-1 interacting protein 1 (LRRFIP-1) promotes pancreatic cancer progression by decreasing the phosphorylation of β-catenin [45]. Wnt pathway is also regulated by post-translational modifications of proteins involved at the receptor level. RNF43 has been shown to ubiquitinate the frizzled receptor, targeting it for degradation, thus, inhibiting the Wnt/β-catenin pathway [46]. Compounds, such as docosahexaenoic acid (DHA), eicosapentaenoic acid (EPA) [47], DNA methyltransferase inhibitor 5-azacytidine [48], calcipotriol (vitamin D analogue) [49], and heparan sulfate mimetic PG545 [50], were all shown to decrease pancreatic cancer progression and metastasis by acting on the Wnt/β-catenin axis and inhibiting the activity of β-catenin. Wnt signaling has been shown to inhibit apoptosis in pancreatic cancer cells by increasing the expression of survivin, a member of the Inhibitor of Apoptosis (IAP) gene family [21].

Micro-RNAs are small non-coding RNAs, which regulate the expression of a plethora of genes, many of which are involved in the hallmarks of cancer. Dysregulated miRNA expression is implicated in the development of different kinds of malignancies, notably pancreatic cancer. Decreased levels of microRNA-195 [51] and miR148a [52] are associated with inhibition of Wnt signaling in pancreatic cancer. MicroRNAs have been linked with a decrease in pancreatic tumor growth by inhibiting the Wnt/β-catenin pathway. For example, miR-454 by downregulating LRP6 [53], miR-940 by decreasing GSK3β and Sfrp1 [54], miR-449a by targeting ataxia-telangiectasia group D complementing gene (ATDC) [55], and miR-300 by targeting CUL4B [56]. In contrast, miR-774 targets the negative regulators of the Wnt/β-catenin axis, such as SFRP1, GSK3β, and TLE3, promoting the stem cell phenotype of pancreatic cancer cells in vitro [57]. Increased expression of long noncoding RNA LINC01133 is observed in pancreatic cancer, where it decreases the expression of DKK1 and increases the expression of Wnt5a, MMP-7, and β-catenin by binding to their promoters [58]. Similarly, the expression of long noncoding RNA H19, which is shown to activate the Wnt pathway, is increased in pancreatic cancer. Besides, miR-194 has been shown to downregulate H19 expression, reversing the effect [59].

Although there is overwhelming evidence indicating that increased β-catenin activity promotes the formation and progression of pancreatic cancer, high expression of PROX1 and β-catenin has been associated with a lower risk of death in PDAC patients in one study [60].

Though there are many well-established mechanisms describing the role of Wnt signaling in pancreatic cancer, there are many unexplored areas. For example, polyamines are upregulated in pancreatic precursor lesions and associated with increased tumor progression [61]. Ornithine decarboxylase (ODC) is the key rate-limiting enzyme in the polyamine synthesis pathway. ODC inhibitor DFMO [62] has been shown to suppress the pancreatic cancer progression by modulating ODC signaling, altering Wnt signaling, decreasing proliferation, and increasing cell cycle arrest markers p53 and p21 [61].

## 3. Inhibitors for Wnt/β-Catenin Signaling Pathway 

Targeting the Wnt/β-catenin signaling pathway is an actively-pursued strategy in the treatment of pancreatic cancer. Several novel inhibitors for Wnt/β-catenin signaling pathway have been developed [16], with their targets and current clinical status summarized in Table 1.

### 3.1. Porcupine Inhibitors 

The porcupine enzyme is required for the secretion of Wnt ligands [63], making porcupine an exciting molecular target of interest. Porcupine inhibitors have been developed to treat Wnt-driven cancers. When porcupine inhibitor LGK974 was screened in 39 pancreatic cancer cell lines, all cell lines carrying inactivating mutations of RNF43 showed sensitivity to LKG974. LGK974 inhibited proliferation and induced differentiation in RNF43-mutant PDAC xenograft model [64]. LKG974 is currently being evaluated in phase I clinical trial (NCT01351103) in pancreatic cancer and other malignancies (Table 1). Another potent porcupine inhibitor ETC-1922159 was shown to be effective in treating RSPO3-translocated colorectal cancer patient-derived xenografts (PDX). Treatment of ETC-19221159 in pancreatic cancer cell lines showed transcriptome changes, cell cycle arrest, increased differentiation markers, decreased stem cells, and proliferation genes [65,66]. ETC-1922159 is currently being evaluated in phase I clinical trial (NCT02521844) in advanced solid tumors (Table 1).

### 3.2. Antibodies Against Wnt Family Proteins

Specific Wnt ligands are overexpressed in many tumors, and monoclonal antibodies are developed against ligands to block their oncogenic activity [16].

#### 3.2.1. OMP-18R5 (Vantictumab) 

OMP-18R5 inhibits canonical Wnt signaling by blocking the binding of Wnt ligands to frizzled receptors 1, 2, 5, 7, and 8 [67]. OMP-18R5 showed growth inhibition in breast, pancreatic, colon, and lung cancer xenograft models. Combination of OMP-18R5 with gemcitabine showed synergism in the pancreatic xenograft model [68]. OMP-18R5 is currently being evaluated in phase I clinical trial in many cancers (Table 1). In a phase Ib study, OMP-18R5 (≥0.5 mg/kg every 2 weeks) in combination with nab-paclitaxel (125 mg/m^2^) and gemcitabine (1000 mg/m^2^) was given on days 1, 8, and 15 of 28-day cycles in 19 previously untreated stage IV pancreatic cancer patients. After observing grade 2 fragility fractures in the first eight patients, subsequent patients were treated with a revised safety plan. Out of 15 toxicity-evaluable patients, 53% (8/15) showed a partial response, and 27% (4/15) were reported with stable disease. The median progression-free survival (PFS) for this cohort was 7.2 months, 95% CI (1.8, 9.2) (9/19 with PFS events) [69].

#### 3.2.2. OMP-54 F28 (Ipafricept)

Fzd8 is a member of the frizzled family of receptors. Ipafricept, a recombinant fusion protein comprised of Fzd8 fused with human immunoglobulin Fc domain, antagonizes Wnt signaling by blocking the binding of membrane-bound frizzled receptors to Wnt proteins [70]. Ipafricept showed significant tumor growth inhibition in pancreatic cancer models when used as a single agent. The addition of paclitaxel markedly increased ipafricept antitumor activity in pancreatic PDX models [71]. In a phase 1b study (2016), 19 patients with previously untreated stage IV pancreatic cancer were treated with ipafricept (≥0.5 mg/kg every 2 weeks) in combination with nab-paclitaxel (125 mg/m^2^) and gemcitabine (1000 mg/m^2^) on days 1, 8, and 15 of 28-day cycles. Out of 14 evaluable patients, 29% (4/14) showed a partial response, and 50% (7/14) were reported with stable disease. PFS was 3.9 months (95% CI (1.7, 7.5)), with 7/19 patients having PFS events [72]. In a phase 1b study (2019), 26 evaluable pancreatic cancer patients were treated with ipafricept in combination with nab-paclitaxel and gemcitabine. Outcomes included 34.6% (9/26) patients with a partial response and 46.2% (12/26) patients showing stable disease, with a clinical benefit rate of 80.8. At the end of the study, PFS was 5.9 months (95% CI (3.4, 18.4)), and median overall survival was 9.7 months (95% CI (7.0, 14)) [73]. These results show that ipafricept could be safely administered in pancreatic cancer patients.

#### 3.2.3. Other Antibodies

OTSA101 is an antibody-drug that targets a novel tumor-specific antigen FZD10 [74]. DKN-01 is a monoclonal antibody against Wnt antagonist DKK1, shown to possess potential anti-osteolytic activity in one study [75]. Both these antibodies are being evaluated in phase I clinical trials (Table 1). 

### 3.3. β-Catenin Inhibitors

#### 3.3.1. PRI-724 

β-catenin binds to co-activators, such as CBP, to activate transcription of a broad range of target genes. PRI-724 inhibits the interaction between β-catenin and CBP. In pancreatic cancer cell lines, PRI-724 promotes differentiation of chemotherapy-resistant cancer stem cells and decreases metastatic potential [76]. In a phase I clinical trial, 20 pancreatic cancer patients (median age = 60 years with 51–73 range) who received first-line treatment with FOLFIRINOX were evaluated. The study design used three dose-cohorts (PRI-724 at 320, 640, and 905 mg/m^2^/d, continuous infusion, 7 days every other week) each in combination with gemcitabine (1000 mg/m^2^ on days 1, 8, 15 of 28 days cycle). Stable disease (by RECIST criteria) was observed in eight of these patients (40%; 8/20), including two with minor responses. Five out of eight patients (62.5%) with elevated CA19-9 baseline levels showed a decline greater than 30%. PFS was 2 months (0.7 to 7.7) [77]. These results indicate PRI-724 combined with gemcitabine is safe to administer in PDAC patients.

Another CBP inhibitor ICG-001 was shown to inhibit both anchorage-dependent and independent growth in multiple PDAC cell lines. ICG-001 altered the expression of several genes involved in DNA replication and cell cycle progression, and significantly increased survival in an orthotopic PDAC xenograft model [34]. ICG-001 was reported to induce cytotoxic effects independent of canonical Wnt signaling inhibition, by transcriptional upregulation of pro-apoptotic proteins Noxa and Puma. ICG-001 has demonstrated anti-tumor effects in several tumor types [78].

#### 3.3.2. Wnt5a Mimetics 

Wnt5a is reported to have a tumor-suppressive function in colon cancer, neuroblastoma, breast carcinomas, and leukemia. However, Wnt5a was shown to be upregulated in pancreatic intraepithelial neoplasia lesions and invasive pancreatic cancers. Similar findings were reported in gastric cancer, melanoma, and lung cancer [79]. Foxy-5, a Wnt5a-mimicking peptide, effectively reduced the metastasis in Wnt5a-low prostate cancer, breast cancer, etc. [80]. It is currently being evaluated in phase I clinical trials (Table 1). 

## 4. Wnt/β-Catenin Signaling in Pancreatic Cancer Drug Resistance

Wnt signaling drives drug resistance in pancreatic cancer in many ways. Cancer stem cells (CSCs) survive after therapy and promote cancer relapse and therapeutic resistance. CSCs regulate drug resistance by elevating expression of ATP-binding cassette transporters, evading cell death, alerting detoxification enzymes like aldehyde dehydrogenases (ALDH), regulating EMT, and escaping from immune surveillance [12]. Wnt signaling and the nuclear functions of β-catenin have been reported to be critical for CSCs proliferation, differentiation, and maintenance [81]. 

Side population is a subset of CSCs that has a high capability for effluxing drugs and has increased metastatic properties. Pancreatic cancer cell lines were shown to harbor side population cells. Side population cells showed Wnt signaling activation and were reported to be involved in resistance to drugs, including 5-FU and irinotecan [82]. Inhibition of Wnt signaling decreased side population cells and increased sensitivity to paclitaxel and irinotecan in cancer cells [82,83]. Culturing pancreatic cells with increasing concentrations of gemcitabine increased the side population, drug efflux, and CSCs markers. The authors concluded that this could be a mechanism contributing to gemcitabine resistance [84]. Pancreatic cancer cells surviving a high dose of gemcitabine were shown to express increased levels of CSCs genes and acquire EMT characteristics [85].

In another study, gemcitabine treatment resulted in elevated expression of EMT genes in pancreatic xenograft models. Treatment with Wnt antagonist OMP-18R5 stimulated downregulation of pancreatic lineage genes AXIN2 and SPP1 in this report. Combination of OMP-18R5 with gemcitabine resulted in delayed tumor growth and diminished the increase in expression of EMT genes, which are responsible for metastasis. Further, the tumor-initiating frequency (TIC), the average number of cells required to cause tumor growth in the recipient cohort, was significantly decreased in the group treated with a combination of gemcitabine and OMP-18R5 compared to other groups [68]. Drugs that target the non-Wnt pathway, such as masitinib (a tyrosine kinase inhibitor), increased pancreatic cancer cell line sensitivity to gemcitabine, through downregulation of the Wnt/β-catenin pathway. Similar results were observed in patients during a phase II clinical study [86]. Wnt5a mediates resistance to gemcitabine in pancreatic cancer cell lines by inducing a transcriptional signaling cascade dependent on NFATc2 in this study [87]. Also, Wnt5a was shown to contribute to chemoresistance by regulating the cell cycle proteins cyclin D1, retinoblastoma protein (pRb), and pRb-E2F complex formation, thereby disturbing G1/S cell cycle progression [88].

Along with their role in pancreatic cancer carcinogenesis, metastasis, and prognosis, miRNAs have been reported to promote pancreatic cancer drug resistance via the Wnt signaling pathway. MiR-29a was shown to induce gemcitabine chemoresistance via the Wnt/β-catenin signaling pathway [89]. MiR-33a was reported to play a role in tumor suppression, where it increased gemcitabine sensitivity in human pancreatic cancer cells by downregulating the nuclear translocation of β-catenin [90]. These results indicate that Wnt signaling regulates gemcitabine resistance in multiple ways, and inhibiting Wnt signaling can reverse the chemoresistance of gemcitabine in pancreatic cancer. 

Wnt antagonists were used to effectively sensitize other drug treatments in pancreatic cancer models. Taxane treatment as a monotherapy was associated with enrichment for tumor cells with high Wnt/β-catenin pathway activity and tumorigenicity. However, sequential dosing of WNT antagonists (vantictumab and ipafricept), followed by taxane treatment, produced superior antitumor efficacy. This effect was coupled with mitotic cell death and promoted cell death in PDX models [71]. These results show that blocking Wnt signaling decreases taxane-resistant cells and can increase the sensitivity of taxane treatment in patients with pancreatic cancer.

Wnt/β-catenin signaling is significantly associated with 5-FU resistance in pancreatic cancer cells. Glypican-4 (GPC4), a member of the glypican family, was shown to regulate Wnt/β-catenin signaling in one study. Inhibition of GPC4 in pancreatic cancer cells enhanced sensitivity to 5-FU, and attenuated stem cell-like properties via suppression of Wnt/β-catenin pathway [91]. 

Irinotecan and liposomal irinotecan (MM-398) [92] have recently been approved for the treatment of pancreatic cancer [93,94]. Oncogenic receptor ErbB2 was shown to modulate gemcitabine and irinotecan/SN-38 chemoresistance in human pancreatic cancer cells via the hCNT1 transporter. Multidrug-resistance in these cells was associated with protein MRP-2 [95]. Wnt signaling appears to regulate both ErbB2 and MRP2 [96,97]. Therefore, Wnt signaling may influence irinotecan drug resistance in pancreatic cancer.

ATP-binding cassette transporter family members have been implicated in pancreatic cancer cell resistance to oxaliplatin [98] and cisplatin [99]. Besides, ATP-binding cassette transporter proteins may be regulated by Wnt signaling [97]. Further, Wnt-signaling members, DKK1 [100] and LGR6 [101] have been shown to modulate cisplatin drug-resistance in other cancer cell lines. 

HDAC inhibitors are FDA approved to treat lymphoid cancers [102,103,104]. Inhibition of the Wnt-signaling pathway reversed resistance to the HDAC inhibitor trichostatin A in pancreatic cancer cells [105]. These results demonstrate that Wnt signaling regulates pancreatic cancer cell resistance to a wide range of drugs. Moreover, these studies highlight the fact that Wnt signaling not only causes chemoresistance in pancreatic cancer but can also influence cellular chemosensitivity to other drugs. 

## 5. Challenges of Targeting the Wnt Pathway

Wnt signaling controls myriad key cellular processes and somatic stem cell maintenance [12] and is involved in normal embryonic development and maintains homeostasis throughout the life in virtually every tissue [13]. Since Wnt signaling is shared by normal and cancer cells in the body, side effects associated with targeting the Wnt pathway are difficult to control. Toxicities related to blood, bone, and gastrointestinal tract are anticipated with the abrogation of Wnt signaling [106]. Administration of vantictumab in pancreatic cancer patients caused significant adverse events, including gastrointestinal side effects and fragility fractures [67,106]. A similar adverse event profile was reported in the phase I trial of ipafricept, where side effects, such as neutropenia (three grade 3 and one grade 4) and hypophosphatemia (one grade 3), were reported in ovarian cancer patients [107]. Seven grade 3 or 4 toxicities were reported in pancreatic cancer patients in the PRI-724 clinical trial [77]. Since pancreatic cancer is driven by various mutations and signaling-factor dysregulation [2,108], multiple combination therapies are required to target pancreatic cancer. Though Wnt inhibitors have acceptable toxicity profiles, side effects will undoubtedly be more pronounced with combination therapy. Although FDA-approved agents, such as niclosamide, sulindac, and pyrvinium, and investigational agents like vitamin D and curcumin were shown to inhibit Wnt signaling [12,109,110], they also affect multiple signaling pathways. Their cytotoxicity observed with these agents might be independent of Wnt signaling. Wnt5a-dependent β-catenin signaling was shown to facilitate metastasis in pancreatic cancer and melanoma, but, in other cancers, it was shown to have contrasting results [80].

## 6. Conclusion

Pancreatic cancer has dismal survival rates, and the Wnt/β-catenin signaling pathway is involved in pancreatic carcinogenesis and therapeutic resistance. Therefore, studies that target Wnt signaling have great potential for therapeutic intervention. However, well-controlled studies designed to improve understanding of the role of Wnt signaling and robust preclinical drug testing of Wnt inhibitors as single agents and combinations in pancreatic cancer are required.

## Figures and Tables

**Table 1:** Inhibitors for Wnt/β-catenin signaling pathway in clinical trials.

Compound	Target	Current Clinical Status
LGK974/WNT974	Porcupine inhibitor	Phase I (NCT01351103)—malignancies dependent on Wnt ligands, including pancreatic adenocarcinoma. Phase II (NCT02649530)—metastatic head and neck squamous cell carcinoma
ETC-1922159	Porcupine inhibitor	Phase I (NCT02521844)—advanced solid tumors
OMP-18R5 (Vantictumab)	Frizzled receptor	Phase I—solid tumors (NCT01345201), phase I (NCT01973309)—combination with paclitaxel in locally recurrent or metastatic breast cancer, phase 1 (NCT01957007)—combination with docetaxel in non-small cell lung cancer, phase I (NCT02005315)—combination with nab-paclitaxel and gemcitabine in stage IV pancreatic cancer
OMP-54 F28 (Ipafricept)	Fzd8-Fc fusion protein	Phase I (NCT01608867)—solid tumors, phase Ib (NCT02069145)—combination with sorafenib in hepatocellular cancer, phase 1(NCT02092363)—combination with paclitaxel and carboplatin in recurrent platinum-sensitive ovarian cancer, phase I (NCT02050178)—combination with Nab-paclitaxel and gemcitabine stage IV pancreatic cancer
OTSA-101	FZD10 mAb	Phase I (NCT01469975)—synovial sarcoma
DKN-01	DKK, dickkopf-related protein	Phase I/II (NCT03645980)—advanced liver cancer, Phase I (NCT01711671)—DKN-01 and lenalidomide/dexamethasone in relapsed or refractory multiple myeloma, Phase I (NCT01457417)—advanced solid tumors, Phase I (NCT02013154)—combination with paclitaxel or pembrolizumab in relapsed or refractory esophagogastric malignancies, Phase I (NCT02375880)—combination with gemcitabine and cisplatin in patients with carcinoma primary to the intra- or extra-hepatic biliary system or gallbladder, Phase II (NCT03395080)—monotherapy or in combination with paclitaxel in recurrent ovarian cancer
PRI-724	β-catenin/CBP	Phase I/II (NCT01606579)—myeloid leukemia, Phase I (NCT01302405)—advanced solid tumors, Phase I (NCT01764477)—combination with gemcitabine in advanced or metastatic pancreatic adenocarcinoma
CWP232291	β-catenin	Phase I/II (NCT03055286 and NCT01398462)—acute myeloid leukemia, Phase I (NCT02426723)—relapsed or refractory myeloma
Foxy-5	Wnt5a	Phase I (NCT02020291)—anti-tumor activity and PK profiles in metastatic breast, colon, or prostate cancer

Inhibitors for Wnt/β-catenin signaling pathway. The clinical trials information was obtained from https://clinicaltrials.gov/, accessed on 8 May 2019.

## Abbreviations

PDAC	Pancreatic ductal adenocarcinoma
EMT	Epithelial mesenchymal transition
LRP	Lipoprotein receptor related protein
DKK1	Dickkopf 1
Dsh	Disheveled
CBP	CREB binding protein
PCP	Planar cell polarity
ODC	Ornithine decarboxylase
OMP	18R5 Vantictumab free survival
PFS	Median progression
OMP	54 F28 Ipafricept
CSCs	Cancer stem cells
